# The IAT shows no evidence for Kandinsky's color-shape associations

**DOI:** 10.3389/fpsyg.2013.00616

**Published:** 2013-09-11

**Authors:** Alexis D. J. Makin, Sophie M. Wuerger

**Affiliations:** Department of Experimental Psychology, University of LiverpoolLiverpool, UK

**Keywords:** shape, form, color, Kandinsky, implicit association test, bauhaus, synesthesia

## Abstract

In the early twentieth century, the Bauhaus revolutionized art and design by using simple colors and forms. Wassily Kandinsky was especially interested in the relationship of these two visual attributes and postulated a fundamental correspondence between color and form: yellow triangle, red square and blue circle. Subsequent empirical studies used preference judgments to test Kandinsky's original color-form combinations, usually yielding inconsistent results. We have set out to test the validity of these postulated associations by using the Implicit Association Test. Participants pressed one of two buttons on each trial. On some trials they classified shapes (e.g., circle or triangle). On interleaved trials they classified colors (e.g., blue or yellow). Response times should theoretically be faster when the button mapping follows Kandinsky's associations: For example, when the left key is used to report blue or circle and the right is used for yellow and triangle, than when the response mapping is the opposite of this (blue or triangle, yellow or circle). Our findings suggest that there is no implicit association between the original color-form combinations. Of the three combinations we tested, there was only a marginal effect in one case. It can be concluded that the IAT does not support Kandinsky's postulated color-form associations, and that these are probably not a universal property of the visual system.

## Introduction

In recent years, there has been a growing interest in the links between art and visual perception, with new specialized journals and conferences. It is recognized that artists might be experts at exploiting the visual system; with special sensitivity to its constraints and parameters, and that vision scientists might learn something by studying paintings in detail (Ramachandran and Hirstein, 1999; Zeki, 2002; Van de Cruys and Wagemans, 2011; Shimamura, 2012).

Wassily Kandinsky (1866–1944) was an influential Russian painter. As his career progressed, Kandinsky produced increasingly abstract images, and for a period from 1922–1933 he taught at the famous Bauhaus school in Germany, which celebrated simple colors and forms. Kandinsky was a theorist as well as an artist, and he derived profound, spiritual meaning from aesthetic experiences. One of Kandinsky's ideas was that there are certain fundamental associations between colors and shapes (Kandinsky, 1912): he proposed *Yellow-Triangle, Blue-Circle, and Red-Square*. These associations were formulated introspectively, however, he did conduct his own survey at the Bauhaus in 1923 by distributing questionnaires to his professorial colleagues and students, and found that many of his colleagues agreed with his associations; notable exceptions were his contemporaries, Klee and Schlemmer, who favored different form-color combinations (Duechting, 1996). In fact, Kandinsky had already embarked upon a similar attempt to identify color form associations while still in Russia with the aim to provide the scientific underpinning for his own intuitions (Poling, 1984).

Recently, the idea of systematic color-form associations has become known as *correspondence theory* (Jacobsen, 2002; Kharkhurin, 2012). It probably crystallized somewhat after Kandinsky's death, becoming more tightly associated with the Bauhaus through various historical accidents. For example, a famous poster for the Bauhaus exhibition in Stuttgart, 1968, designed by Herbert Bayer, showed the yellow triangle, red square, and blue circle (Jacobsen and Wolsdorff, 2007).

After Kandinsky's original survey in 1923, evidence for correspondence theory has been limited. Jacobsen (2002) administered a modified version of Kandinsky's questionnaire to a sample of non-artist university students, half of whom were asked to choose combinations of colors and shapes (mere correspondence), and half were asked which combinations they found aesthetically pleasing (aesthetic correspondence). It was found that pragmatic associations influenced participant's choices, for example, they typically paired red with triangle because of association with traffic signs, and yellow with circle because this looked like the sun. Moreover, these subjects actually disliked the combinations devised by Kandinsky compared to other options. More recently, Jacobsen and Wolsdorff (2007) found that a sample of art experts had their own color-form combination preferences, but these were again in disagreement with correspondence theory.

Most recently, Albertazzi et al. (2013) asked participants to choose the color that they felt naturally went with each of 12 shapes, including some 3D shapes. Participants were explicitly told to avoid associations from memory, of the type reported by Jacobsen (2002). If people have no systematic color form associations, then all combinations would appear equally. However, some combinations were chosen significantly more often than others. The results were partially in agreement with Kandinsky's correspondence theory (Yellow and Triangle, Red and Square), but there was little evidence for blue and circle associations, and several other associations, not discussed by Kandinsky, were also reported.

The topic has also been explored by Holmes and Zanker (2008) who used an innovative oculomotor evolutionary algorithm method. Virtual “genes” controlled stimulus characteristics such as color and shape. For example, for one member of the starting population, the color gene might be set to yellow, and the shape gene set to square, so the individual stimulus would be a yellow square. For another stimulus the color and shape genes would code a different combination. At the beginning of the experiment, the computer generated a “population” of many such stimuli, with randomly chosen virtual genes. On each trial, a subset of the starting population would be shown on the screen, and the participants looked for the ones they like most. Genes were assigned a fitness score based on feedback from an eye tracker. After a certain number of trials, the stimuli entered a competitive tournament, and only the stronger combinations were passed on to the next generation. Over the trials, stimuli evolved to become more like those that best attract participant's gaze. However, while consistent preferences were found within each individual, no systematic color-form combinations emerged across observers.

Kandinsky may have had an intriguing condition known as synesthesia, where multimodal connections create idiosyncratic, additional perceptual experiences. For example, in color-grapheme synesthetes, who have been best studied, the letter E might be perceived as if it is always written in red ink. Synesthesia does not reflect trivial associations and beliefs: grapheme-color synesthetes show improved letter detection performance in visual search tasks, and, in some subjects, the color-sensitive brain area V4 is activated by presentation of black and white graphemes (Hubbard and Ramachandran, 2005). Many theorists cited above have suggested that Kandinsky's synesthesia may have inspired correspondence theory.

Given the research to date, it is entirely possible that correspondence theory says something about Kandinsky's personal artistic elaborations or the socially constructed aesthetics of the Bauhaus movement, but says nothing about the architecture of the average person's visual system. However, Hubbard and Ramachandran (2005) suggest that mechanisms present in synesthetes may be present to a *lesser degree* in non-synesthetes, and this could account for the “conceptual rightness” of certain, widely held multisensory mappings, such as the Bouba–Kiki effect, where jagged or bulbous visual shapes seem to “go” with certain sounds (in this case, “Bouba” with rounded shapes, “Kiki” with spiky shapes). Moreover, while synesthetic associations are strikingly idiosyncratic, most people sense the Bouba–Kiki associations. Correspondence theory could reflect Kandinsky's intuition into some of these sub-clinical, quasi-synesthetic pairings, which, like the Bouba–Kiki effect, are near-universal.

It is possible that these quasi-synesthetic associations might not be explicitly recognized, but affect perceptual judgments nevertheless. Following this reasoning, Kharkhurin (2012) evaluated correspondence theory with an implicit priming experiment. Participants were shown a colored screen for 1 s, then a shape, which they had to classify as quickly as possible as triangle, square or circle. The hypothesis was that people would classify the shape quicker on congruent trials, where the prime color was associated with the shape according to correspondence theory. In a second experiment, this was reversed, and shapes were used as a prime before color classification. Neither experiment found any facilitation of reaction time in the congruent trials, so this study failed to provide any empirical support for correspondence theory using implicit priming techniques.

Kharkhurin's (2012) work was based on a priming paradigm, which, although valid and potentially informative, may not have been optimized for detecting associations between two visual dimensions. In Kharkhurin's study, the primes were task-irrelevant, and could potentially be ignored completely, while the targets were classified very easily. Recent work on affective priming and symmetry has found this technique to be much weaker than different paradigms where participants are forced to classify all stimuli (Bertamini et al., 2013a). In the current work, we test correspondence theory using the Implicit Association Test (IAT, Greenwald et al., 1998; Nosek et al., 2007), which has been used in thousands of experiments, often revealing associations between dimensions which people are either explicitly unaware of, or even explicitly reject. Recently, the IAT has been used to answer various questions in empirical aesthetics (Gattol et al., 2011; Mastandrea et al., 2011; Makin et al., 2012a,b; Bertamini et al., 2013b). Importantly, the IAT procedure requires participants to classify all stimuli. Furthermore, it was specifically designed to probe associations between stimulus pairs, and it has been subjected to extensive methodological scrutiny (Nosek et al., 2007).

The best way to describe the IAT is through example. In one of the best-known IAT experiments, participants were given two buttons, which were used to classify four stimulus categories (Greenwald et al., 1998). On some trials they saw pictures of either flowers or insects, and had to press one button for flower and the other button for insect. On interleaved trials, they saw either positive words (e.g., LOVE) or negative words (e.g., HATE). They had to press one button for positive, and the other for negative. In *congruent blocks*, the same button was used to report a flower or positive word, and the other was used to report insect or negative word. In *incongruent blocks*, the response mapping was reversed (so one button was used to report flower and negative, the other was used to report insect and positive). Because people usually associate flowers with other positive things and insects with negative things, the task was much harder in the incongruent block, and reaction times were therefore longer. The existence of an RT difference between congruent and incongruent blocks can be taken as evidence for an implicit association between the stimulus pairs. In this example, the implicit associations measured by the IAT were in agreement with the explicitly held attitudes of the participants, who preferred flowers to insects.

In another experiment, Greenwald et al. (1998) used the IAT to reveal implicit racial prejudices in participants who were not overtly racist. White participants were quicker to respond in congruent blocks, where white faces and positive words were reported with the same key, and black faces and negative words were reported with the other key, then in incongruent blocks, where the response mapping was reversed (white and negative, black and positive). Given that the IAT is sensitive to hidden or unconscious associations, it might reveal color-form correspondences that are not explicitly acknowledged.

In this work we presented 36 participants with three separate IAT experiments in a within-subjects design (see Gattol et al., 2011 for another example of this “multidimensional” IAT approach). Each IAT experiment compared two colors and two shapes. In the congruent blocks, color-form mapping was in line with Kandinsky's correspondence theory (yellow-triangle, blue-circle, red-square). Let's consider IAT 1 for example: in the congruent blocks participants had to press the left key for blue or circle, and the right key for yellow or triangle (both Kandinsky's correspondences). In the incongruent blocks, response mapping was the opposite of correspondence theory, so the left key would be used for blue or triangle and the right key would be used for yellow or circle (Opposite of Kandinsky's correspondences). Tables 1 and 2 show the structure of IAT 1 in more detail. The three IAT experiments cover every combination of color and shape proposed by correspondence theory (Table 3).

**Table 1:** **Order of blocks and response mappings for participants who did congruent trials first (example from IAT 1)**.

**Block**		***N* trials**	**Left key**	**Right key**
1	Training 1	20	Circle	Triangle
2	Training 2	20	Blue	Yellow
3	Congruent 1	20	Circle or Blue	Triangle or Yellow
4	Congruent 2	40	Circle or Blue	Triangle or Yellow
5	Training 3	20	Triangle	Circle
6	Training 4	20	Triangle	Circle
7	Incongruent 1	20	Triangle or Blue	Circle or Yellow
8	Incongruent 2	40	Triangle or Blue	Circle or Yellow

**Table 2:** **Order of blocks and response mappings for participants who did incongruent trials first (example from IAT 1)**.

**Block**		***N* trials**	**Left key**	**Right key**
1	Training 1	20	Triangle	Circle
2	Training 2	20	Blue	Yellow
3	Incongruent 1	20	Triangle or Blue	Circle or Yellow
4	Incongruent 2	40	Triangle or Blue	Circle or Yellow
5	Training 3	20	Circle	Triangle
6	Training 4	20	Circle	Triangle
7	Congruent 1	20	Circle or Blue	Triangle or Yellow
8	Congruent 2	40	Circle or Blue	Triangle or Yellow

**Table 3:** **The response mappings in our 3 IAT experiments**.

	**IAT 1**		**IAT 2**		**IAT 3**	
	**Left key**	**Right key**	**Left key**	**Right key**	**Left key**	**Right key**
**Congruent blocks**	Circle	Triangle	Circle	Square	Square	Triangle
	Blue	Yellow	Blue	Red	Red	Yellow
**Incongruent block**	Triangle	Circle	Square	Circle	Triangle	Square
	Blue	Yellow	Blue	Red	Red	Yellow

## Method

### Participants

Thirty-six participants were involved (age 18–45, 11 male, 6 left handed). Most were involved with undergraduate or postgraduate study at the University of Liverpool. All had normal or corrected to-normal vision. The male participants were checked for color blindness using the 1966 Ishihara plates (Ishihara, 1917), and none were aware of the Kandinsky color form correspondence theory (or were at least unaware of the exact combinations he proposed).

### Apparatus and stimuli

**Figure 1:**
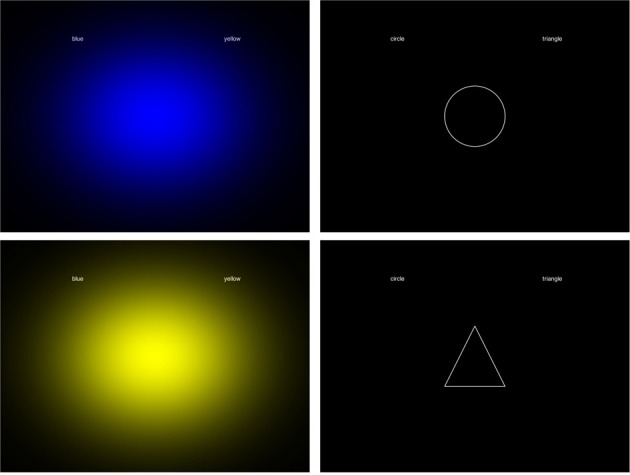
**Example stimuli used in the current experiment**.

Stimuli were presented at ~57 cm on a 1280 × 1024 pixel CRT monitor, with a refresh rate of 60 Hz. Stimuli were generated using open source Psychopy software (Peirce, 2007). Participants entered responses using the left [A] and right [L] keys of a standard computer keyboard. The three shapes were white line drawings on a black background. The circle was 6.3° in diameter, the square was 6.3 × 6.3°, and the triangle was 6.3° tall, and 6.3° wide, always presented with the same upwards orientation. Color patches were ~20° wide (Gaussian-modulated full screen, Figure 1). Since there are no precise records of the colors used by Kandinsky, we used a set of primary colors (red, yellow, blue) close to unique hues as judged by a color-normal experienced observer (Wuerger et al., 2005; Wuerger, 2013). The CIE coordinates and the luminance of the colored Gaussian patches were as follows: yellow *x* = 0.406; *y* = 0.512; luminance = 59 cd/m^2^; red: *x* = 0.628; *y* = 0.331; luminance = 14 cd/m^2^; blue: *x* = 0.152; *y* = 0.071; luminance = 8 cd/m^2^. Example stimuli from IAT 1 are shown in Figure 1.

### Procedure

Each participant completed 3 IAT experiments, which took about 7 min each. The structure of a single IAT experiment was based on the recommendations of Nosek et al. (2007). There were 8-blocks in total. Half the participants did the congruent blocks first. For these participants, the IAT 1 would have run as shown in Table 1. The first block was a training block, where participants discriminated shape only (e.g., left for circle, right for triangle). The second block was another training block, where participants discriminated color only (e.g., left button for blue, right for yellow). The third and fourth *congruent blocks* combined color and shape discrimination, and the response mapping fitted Kandinsky's theory (e.g., left for circle or blue, right for triangle or yellow). Next two further training blocks were given, where participants had to relearn the key mapping for shapes (e.g., left button for triangle, right for circle). Finally, two incongruent blocks were presented, where the response mapping was the opposite of Kandinsky's correspondence theory (e.g., left for triangle or blue, right for circle or yellow). The other participants did the incongruent block first, and the training blocks were rearranged accordingly (Table 2). In each block, the trials were presented in a novel random order for each participant. The order in which participants did the three IAT experiments, and the number of participants doing congruent or incongruent blocks first was counterbalanced.

All blocks were preceded by appropriate instructions informing participants of the stimuli and response requirements. When stimuli were presented, cue words reminded participants which keys should be used to report their answers. For example, when a shape appeared in first training block of IAT 1, the words “circle” and “triangle” appeared on the left and right sides of the screen. When participants pressed the wrong button, the word “Wrong” appeared centrally in red, and remained until the correct key was pressed.

## Analysis

Kandinsky's correspondence theory would be supported by faster reaction times in congruent blocks than incongruent blocks. We processed the data according to the recommendations of Nosek et al. (2007), which are widely used in the IAT literature (e.g., Gattol et al., 2011). Training blocks were excluded from analysis. Trials where participants pressed the wrong button were also excluded (6%). For each participant and experiment, the following data processing steps were taken.

1. We then obtained the mean RT in blocks 3,4,7, and 8.
2. We computed the difference in mean RT between blocks 3 and 7, and the difference 4 and 8.
3. We the obtained the standard deviation of response times in blocks 3 and 7. We then divided the difference in mean RT between 3 and 7 by this standard deviation, giving a *D score*.
4. We repeated this procedure for blocks 4 and 8, to give another *D score*.
5. D scores from steps 3 and 4 were averaged, to give a single D score for that participant and IAT.

The D score is the difference between incongruent and congruent blocks in standard deviation units. A positive value means the hypothesis was supported, and negative value means that the participant associates stimuli in the opposite way to that predicted. We got three D scores from each participant: one from each IAT experiment in Table 3. We used one-sample *t*-tests to explore whether D scores across participants were significantly greater than zero. These variables in this analysis were normally distributed according to the Shapiro-Wilk test (*p* > 0.219).

## Results

**Figure 2:**
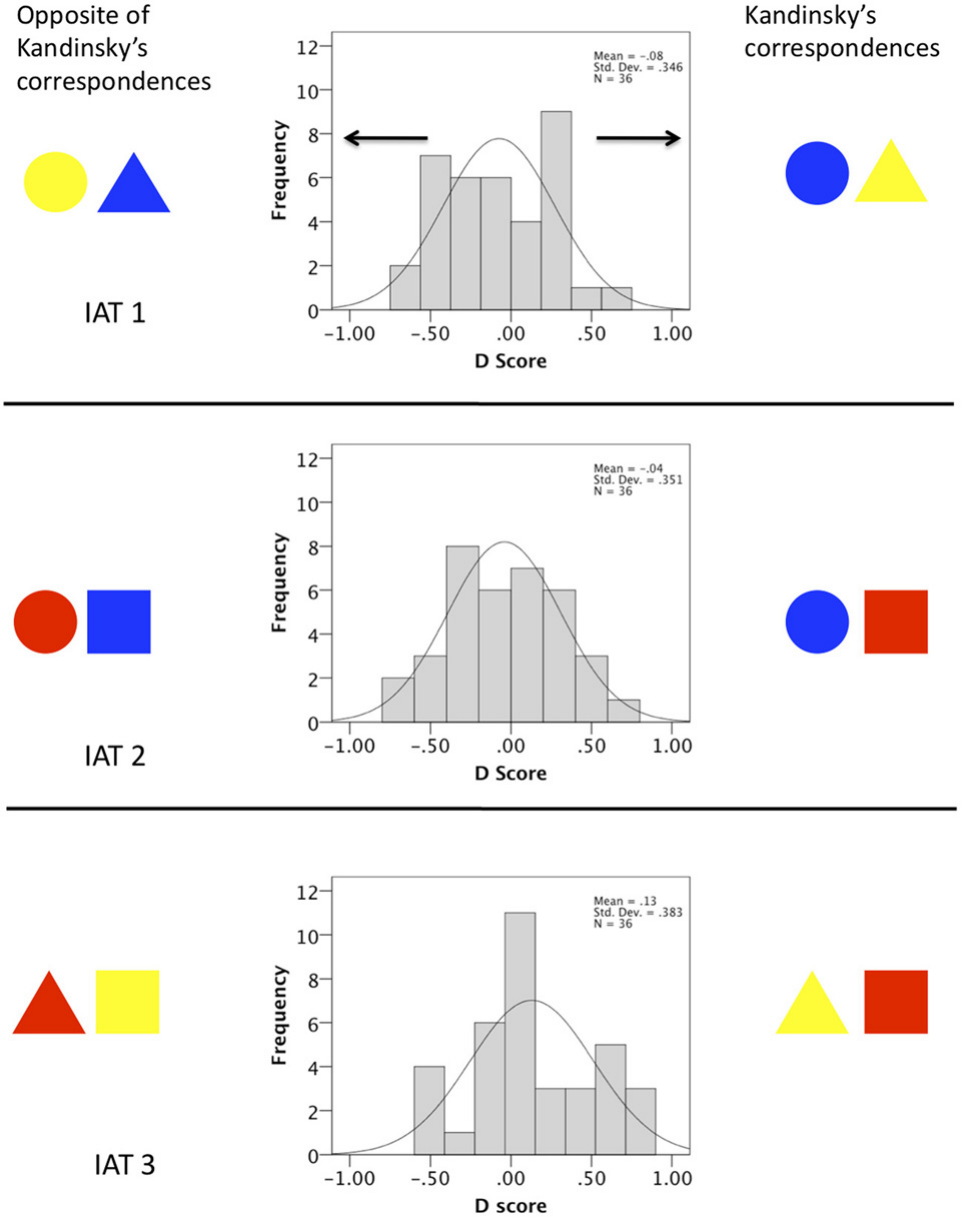
**Results of three IAT experiments designed to measure color-form correspondence theory.** Positive D scores reflect faster performance in congruent blocks. The Histogram shows the number of participants with D scores within a given range. In IATs 1 and 2 the 36 D scores were normally distributed around zero. In IAT 3 there was a marginally significant shift the right, meaning that on average, participants associated yellow with triangle and red with square compared to the opposite, in line with Kandinsky's theory.

Participants completed three IAT experiments, each comparing a pair of Kandinsky's color form correspondences. In the congruent blocks, response mapping was in accordance with the theory, while in the incongruent blocks, response mapping was the opposite of correspondence theory. Faster reaction times in the congruent blocks would support the theory. We found limited evidence for this in our IAT experiments. As shown in Figure 2, D scores were normally distributed around zero in IAT1 [*t*_(35)_ = −1.301, *p* = 0.202] and IAT 2 [*t*_(35)_ = < 1, N.S]. However, In IAT 3 there was a borderline effect in the expected direction in IAT 3 [*t*_(35)_ = 2.020, *p* = 0.051].

Repeated measures ANOVA tested whether there was a difference in the D scores between the three IAT experiments, and whether this interacted with the between-subjects factors of experiment order (the order which the three IAT experiments were presented) and block order (congruent block first or second). There was a borderline significant effect of IAT [*F*_(2, 60)_ = 3.141, *p* = 0.050], because of the slightly larger D score in IAT 3, described above. There were no interactions involving experiment order [*F*_(4, 60)_ < 1, N.S], or block order [*F*_(2, 60)_ = 1.744, *p* = 0.183].

There are various approaches to IAT analysis. Some researchers do not include the first blocks of congruent and incongruent trials (Blocks 3 and 7 in Table 1) and analyze D scores from the longer second blocks only (Blocks 4 and 8). With this approach, the effect in IAT3 was again borderline significant [*t*_(35)_ = 1.969, *p* = 0.057].

Another approach is to simply compare mean response time in congruent and incongruent blocks (without standardization to D scores). In our study, this approach found no effects in IAT1 [609 vs. 588 ms. *t*_(35)_ = −1.241, *p* = 0.223] or IAT2 [630 vs. 601 ms. *t*_(35)_ = −1.502, *p* = 0.142], [575 ms. in the congruent block vs. 621 ms. in the incongruent block, *t*_(35)_ = 2.498, *p* = 0.017].

We next analyzed response times with mixed ANOVA with two within-subject factors [experiment (IAT1, IAT2, or IAT3) × block (congruent or incongruent)] and two between subject factors (3 experiment order × 2 block order). Correspondence theory predicts a main effect of block, resulting from uniform faster responses in the congruent blocks. There was no evidence for this [*F*_(1, 30)_ < 1, N.S]. Furthermore, there was no overall response time difference between the three IAT experiments [*F*_(1, 61, 48.26)_ < 1, N.S.]. There was, however, an experiment × congruence interaction [*F*_(2, 60)_ = 5.517, *p* = 0.006]. This was because of the unique effect of congruence in IAT3, mentioned above. There was also an IAT × experiment order interaction [*F*_(3, 22, 48.26)_ = 3.654, *p* = 0.017], partly because participants tended to take longer to produce responses in their first IAT than the next two. There were no other effects or interactions [next largest *F*_(1, 30)_ = 1.777, *p* = 0.193].

Finally, we predicted that participants should make fewer errors in the congruent blocks. There was no difference in error rates between congruent and incongruent blocks in IAT1 [*t*_(35)_ < 1, N.S.] or IAT2 [*t*_(35)_ < 1, N.S.]. However, responses were more accurate in the congruent blocks of IAT3 [*t*_(35)_ = 3.431, *p* = 0.002]. So, while there was some marginal support for Kandinsky's correspondence theory from IAT 3, we acknowledge most evidence for this effect would disappear completely if we employed correction for multiple comparisons, so we do not consider it instructive without replication.

## Discussion

Our series of three IAT experiments provided no support for Kandinsky's color form correspondence theory. Participants completed three IAT experiments; each designed to compare one pair of color-shape associations with the opposite. In IATs 1 and 2, Reaction times were comparable in congruent and incongruent blocks, suggesting no special associations between particular combinations. These results are thus in agreement with those of Kharkhurin (2012) and Holmes and Zanker (2008), who also found no evidence for correspondence theory using different implicit procedures. In IAT 3, there was a marginally significant effect in the expected direction, suggesting that participant's associate triangle with yellow and square with red, as Kandinsky would have predicted. However, this effect was not strong, and would require replication.

Our IAT experiments did not test whether people have *preference* for yellow triangles, red squares and blue circles over other color-shape pairings. However, we think these putative color shape associations would be theoretically interesting even without aesthetic preferences. For one thing, a clear, positive result in our IAT experiments would suggest that Kandinsky had intuitively recognized an obscure and unexplained property of the visual system, which would require further research. Our null results have different implications: We conclude that Kandinsky's correspondence theory has little to say about the architecture of the typical visual system, and must have other origins. Leading thinkers in scientific aesthetics are probably justified when they claim that artists possess a unique kind of knowledge about human vision (e.g., Zeki, 2002; Van de Cruys and Wagemans, 2011), however, correspondence theory is probably *not* an example of this special insight.

It is very unlikely that the null results reported here can be attributed to low power. There were 36 participants, a much larger sample size than used in other IAT experiments in aesthetic science. For example, Makin et al. (2012a) used the IAT to detect an implicit preference for symmetrical over random patterns with 12 subjects, and replicated this in other experiments with just 6 subjects. The mean D scores in these experiments were around 0.5, whereas the highest D score here was 0.12. Further examples from diverse fields allow this effect size to be seen in context: Gattol et al. (2011) found D scores ranging around 0.2–0.4 for associations between different car brands and dimensions such as “aggressive-peacefulness” and “conventional-innovative,” while Dasgupta et al. (2009) reported D scores of at least 0.5 in a series of IATs measuring implicit attitudes toward social in-groups and out-groups. These are all higher than the largest D score found in IAT3.

Moreover, we conducted three IAT experiments, and did not correct for multiple comparisons. This would increase the chances of a false positive, but we still only found a borderline effect in one of our three experiments. Finally, there was an asymmetry in the design of this experiment, which can be seen in Figure 2: Across the three IATs, a shape was twice as likely to share a button with the color proposed by correspondence theory as any other color (for example, a participant would be presented with two experiments where yellow and triangle were reported with the same button, but only a single experiment where blue and triangle or red triangle shared a button). If anything, this design feature would produce false positives in favor or correspondence theory, which we did not find. This data, in conjunction with previous work, seems to rule out Kandinsky's correspondence theory in any strong form, so we think Kandinsky's theory is very unlikely to constitute an insight into the architecture of the average visual system.

Jacobsen (2002) reported different color-form associations to Kandinsky, which seemed to be based on pragmatic associations. For example, his participants paired yellow with circle, because the sun is a yellow circle. We did not find any further evidence for this with our IAT experiments. However, Jacobsen (2002) used a modified version of Kandinsky's questionnaire, which required people to choose preferred particular color-shape combinations from the available options. Perhaps these pragmatic associations only exist when people are forced to make explicit, verbal judgments? Moreover, a very recent study using an explicit matching task (Albertazzi et al., 2013), found partial support for the correspondence theory; observers had to chose their preferred color-form combination, similar to Kandinsky's questionnaire. The authors report a strong association between a triangle and yellow, some evidence for red being associated with a square, but there was no support for the blue-circle combination.

It seems likely that Kandinsky's correspondence theory was influenced by his theorizing, or perhaps indirectly by his synesthesia, and was then spread by subsequent Bauhaus literature and promotion. It seems unlikely that these color-shape associations reflect a common property of all human brains, akin to sub-threshold synesthesia, or that they are an aesthetic universal. Our null results are important because it is likely that people will continue seeking empirical evidence for Kandinsky's correspondence theory, given the current popularity of empirical aesthetics.

Despite our conclusive negative findings, many associations involving color exist in the human brain, some of which may have an ecological basis. For example, Palmer and Schloss (2010) describe evidence that color preferences arise from associations with pleasant and unpleasant objects (e.g., clear blue sky vs. brown feces or rotting food). More generally, it is well-known that statistical properties of the environment (such as a common motion direction for objects and sound sources) are reflected in the neural mechanisms that combine stimuli from different modalities (e.g., Meyer and Wuerger, 2001; Harrison et al., 2011), and that the typical color of objects complements perception, so that, for example, gray bananas still appear slightly yellow (Hansen et al., 2006). However, evidence for apparently meaningless, quasi-synesthetic, color-form associations of the type proposed by Kandinsky remains limited.

### Conflict of interest statement

The authors declare that the research was conducted in the absence of any commercial or financial relationships that could be construed as a potential conflict of interest.

## References

[B1] Albertazzi L. Da Pos O. Canal L. Micciolo M. Malfatti M. Vescovi M. (2013). The hue of shapes. J. Exp. Psychol. Hum. Percept. Perform. 39, 37–47 10.1037/a0028816 22708741

[B2] Bertamini M. Makin A. D. J. Pecchinenda A. (2013a). Testing whether abstract patterns produce affective responses. PLoS ONE 8:e68403 10.1371/journal.pone.0068403 23840892 PMC3698216

[B3] Bertamini M. Makin A. D. J. Rampone G. (2013b). Implicit association of symmetry with positive valence, high arousal and simplicity. i-Perception 4, 317–327 10.1068/i0601jw

[B4] Duechting H. (1996). Farbe am Bauhaus – Synthese und Synaesthesie. Berlin: Gebr. Mann

[B5] Dasgupta N. DeSteno D. Williams L. A. Hunsinger M. (2009). Fanning the flames of prejudice: the influence of specific incidental emotions on implicit prejudice. Emotion 9, 585–591 10.1037/a0015961 19653784

[B6] Gattol V. Saaksjarvi M. Carbon C.-C. (2011). Extending the implicit association test (IAT): assessing consumer attitudes based on multi-dimensional implicit associations. PLoS ONE 6:e15849 10.1371/journal.pone.0015849 21246037 PMC3016338

[B7] Greenwald A. G. McGhee D. E. Schwartz J. L. K. (1998). Measuring individual differences in implicit cognition: the implicit association test. J. Pers. Soc. Psychol. 74, 1464–1480 10.1037/0022-3514.74.6.1464 9654756

[B8] Hansen T. Olkkonen M. Walter S. Gegenfurtner K. R. (2006). Memory modulates color appearance. Nat. Neurosci. 9, 1367–1368 10.1038/nn1794 17041591

[B9] Harrison N. R. Wuerger S. M. Meyer G. F. (2011). Reaction time facilitation for horizontally moving auditory-visual stimuli. J. Vis. 10, 1–21 10.1167/10.14.16 21163957

[B10] Holmes T. Zanker J. (2008). Bauhaus revisited: identifying form and colour preference using a gaze driven evolutionary algorithm. Perception 37, ECVP Abstract Supplement, 148.

[B11] Hubbard E. M. Ramachandran V. S. (2005). Neurocognitive mechanisms of synesthesia. Neuron 48, 509–520 10.1016/j.neuron.2005.10.012 16269367

[B12] Ishihara S. (1917). Tests for Color-Blindness. Handaya; Tokyo: Hongo Harukicho

[B13] Jacobsen T. (2002). Kandinsky's questionnaire revisited: fundamental correspondence of basic colors and forms? Percept. Mot. Skills 95, 903–913 10.2466/pms.2002.95.3.903 12509195

[B14] Jacobsen T. Wolsdorff C. (2007). Does history affect aesthetic preference? Kandinsky's teaching of colour-form correspondence, empirical aesthetics and the Bauhaus. Des. J. 10, 16–27 10.2752/146069207789271902

[B15] Kandinsky W. (1912). Über das Geistige in der Kunst, VI. Kapitel, Formen und Farbensprache. München: Piper

[B16] Kharkhurin A. V. (2012). Is triangle really yellow? An empirical investigation of Kandinsky's correspondence theory. Empirical Stud. Arts 30, 167–182 10.2190/EM.30.2.d

[B17] Makin A. D. J. Pecchinenda A. Bertamini M. (2012a). Grouping by closure influences subjective regularity and implicit preference. i-Perception 3, 519–527 10.1068/i0538 23145305 PMC3485860

[B18] Makin A. D. J. Pecchinenda A. Bertamini M. (2012b). Implicit affective evaluation of visual symmetry. Emotion 12, 1021–1030 10.1037/a0026924 22251051

[B19] Mastandrea S. Bartoli G. Carrus G. (2011). The automatic aesthetic evaluation of different art and architectural styles. Psychol. Aesthet. Crea. 5, 126–134 10.1037/a0021126

[B20] Meyer G. F. Wuerger S. M. (2001). Crossmodal integration of auditory and visual motion signals. Neuroreport 12, 2557–2560. 11496148 10.1097/00001756-200108080-00053

[B21] Nosek B. A. Greenwald A. G. Banaji M. R. (2007). The implicit association test at age 7: a methodological and conceptual review, in Automatic Processes in Social Thinking and Behavior, ed Bargh J. A. (Philadelphia: Psychology press), 265–292

[B22] Palmer S. E. Schloss K. B. (2010). An ecological valence theory of human color preference. Proc. Natl. Acad. Sci. U.S.A. 107, 8877–8882 10.1073/pnas.0906172107 20421475 PMC2889342

[B23] Peirce J. W. (2007). PsychoPy - Psychophysics software in Python. J. Neurosci. Methods 162, 8–13 10.1016/j.jneumeth.2006.11.017 17254636 PMC2018741

[B24] Poling C. V. (1984). Kandinsky in Russland and am Bauhaus 1915–1933 [Kandinsky: Russiche Zeit und Bauhausjahre 1915–1933]. Ausstellungs katalog Bauhaus-Archiv Berlin, Museum fuer Gestaltung.

[B25] Ramachandran V. S. Hirstein W. (1999). The science of art: a neurological theory of aesthetic experience. J. Concious. Stud. 6, 15–31

[B26] Shimamura A. P. (2012). Towards a science of aesthetics, in Aesthetic Science: Connecting Minds, Brains and Experiences, eds Shimamura A. P. Palmer S. E. (New York, NY: Oxford University Press), 3–28

[B27] Van de Cruys S. Wagemans J. (2011). Putting reward in art: a tentative prediction error account of visual art. i-Perception 2, 1035–1062 10.1068/i0466aap 23145260 PMC3485793

[B28] Wuerger S. M. Atkinson P. Cropper S. (2005). The cone inputs to the unique-hue mechanisms. Vision Res. 45, 3210–3223 10.1016/j.visres.2005.06.016 16087209

[B29] Wuerger S. (2013). Colour constancy across the life span: evidence for compensatory mechanisms. PLoS ONE 8:e63921 10.1371/journal.pone.0063921 23667689 PMC3648508

[B30] Zeki S. (2002). Neural concept formation and art: Dante, Michelangelo, Wagner. J. Concious. Stud. 9, 53–76 10.1142/9781860945915_0002

